# Chest Imaging for Pulmonary TB—An Update

**DOI:** 10.3390/pathogens11020161

**Published:** 2022-01-26

**Authors:** Michael Nel, Zoe Franckling-Smith, Tanyia Pillay, Savvas Andronikou, Heather J. Zar

**Affiliations:** 1 Department of Paediatrics and Child Health, Red Cross War Memorial Children’s Hospital, and The SA-MRC Unit on Child and Adolescent Health, University of Cape Town, Cape Town 8001, South Africa; michaelnel6@gmail.com (M.N.); zoe.franckling-smith@uct.ac.za (Z.F.-S.); 2 Department of Radiology, Chris Hani Baragwanath Academic Hospital, Johannesburg 1864, South Africa; tanyiapillay@gmail.com; 3 Department of Radiology, The Children’s Hospital of Philadelphia, Philadelphia, PA 19104, USA; doctor.andronikou@gmail.com

**Keywords:** tuberculosis, chest imaging, children, X-ray, ultrasound, computed tomography, magnetic resonance imaging

## Abstract

The diagnosis of pulmonary tuberculosis (PTB) in children is challenging. Difficulties in acquiring suitable specimens, pauci-bacillary load, and limitations of current diagnostic methods often make microbiological confirmation difficult. Chest imaging provides an additional diagnostic modality that is frequently used in clinical practice. Chest imaging can also provide insight into treatment response and identify development of disease complications. Despite widespread use, chest radiographs are usually non-specific and have high inter- and intra-observer variability. Other diagnostic imaging modalities such as ultrasound, computed tomography (CT), and magnetic resonance imaging (MRI) can provide additional information to substantiate diagnosis. In this review, we discuss the radiological features of PTB in each modality, highlighting the advantages and limitations of each. We also address newer imaging technologies and potential use.

## 1. Introduction

The diagnosis of pulmonary tuberculosis (PTB) remains a challenge, especially in young children in whom non-specific clinical presentation, difficulty in collecting adequate samples for microbiologic testing, and pauci-bacillary load can result in diagnostic uncertainty. Chest imaging provides a useful tool to support the clinical diagnosis. 

However, chest imaging as a diagnostic tool for paediatric PTB has specific challenges depending on the modality used, including poor inter-observer reliability, non-specific radiological signs, and lack of standardized scoring or classification systems [1]. This paper aims to review the different radiographic modalities and features for diagnosis of paediatric PTB, their use and limitations, as well as newer imaging techniques. 

## 2. Overview of Imaging Techniques Available

Chest radiographs are the primary radiologic investigation in children for diagnosis and assessment of PTB [2,3]. Radiograph findings closely reflect the pathophysiology of the disease (Table 1) [4]. Parenchymal foci associated with lymphadenopathy (Ghon complex) are often small and difficult to identify on chest radiograph [5]. Despite the reliance on radiographs in the diagnosis of paediatric PTB, poor sensitivity, specificity, and wide inter observer agreement have been demonstrated [2,6]. Sensitivity of chest radiography for detecting lymphadenopathy, when compared against CT imaging, has been shown to be 67–74%, with a specificity of 39–59% [1,4,6]. The use of the lateral radiograph in improving sensitivity or specificity is controversial. One study found that a lateral radiograph in addition to a frontal image did not significantly improve the diagnostic yield, but increased cost and radiation exposure [6]. In another study, a moderate correlation between findings of lymphadenopathy on lateral chest radiograph and CT scan was shown, with precarinal lymph nodes associated with the highest sensitivity and specificity [7]. The detection of lymphadenopathy on chest radiographs has significant inter-observer variability (average weighted kappa of 0.33–0.36) and poor intra-observer agreement (average weighted kappa of 0.55) [2,8,9].

A chest ultrasound offers a portable, non-invasive, and real-time assessment of intrathoracic pathology without the use of ionizing radiation. Ultrasound allows trained clinicians to make prompt diagnostic and management decisions at the bedside. A major advantage of ultrasound is the ability to assess for features of extrapulmonary TB including ascites, hepatic micro abscesses, pericardial effusion or abdominal lymph nodes. Ultrasound abnormalities suggestive of PTB (consolidation, pleural effusion, or lymphadenopathy) may be found in 31 to 83% of confirmed cases with high inter-reader agreement [10,11]. Diagnostic accuracy increases with user experience [12]. However, data on the sensitivity and specificity of ultrasound for PTB in children are still scanty. Most studies have compared ultrasound with chest radiograph findings. This makes the assessment of diagnostic accuracy difficult due to the poor accuracy of chest radiographs. The use of clinical diagnosis as a reference standard is similarly flawed. Comparison with gold standard imaging techniques, such as CT scanning, would provide valuable information regarding the usefulness of ultrasound in the diagnosis and management of PTB.

CT scanning is regarded as the “gold standard” for imaging of primary pulmonary TB in children. Cross-sectional images as well as 3D image processing provide excellent anatomical detail and bypass the problem of superimposition of anatomical structures in chest radiographs. CT scanning allows for earlier and more frequent detection of lymphadenopathy, consolidation or pleural effusion when compared with chest radiographs [3,8,13]. Beyond this, CT allows for more accurate assessment of disease process, activity, and detection of complications [14].

CT is particularly good for characterizing lymph nodes. The presence of calcification, although uncommon, within a lymph node is highly suggestive of TB [15]. The pattern of enhancement of lymphadenopathy can distinguish PTB from other diseases. TB nodes typically either ring enhance (indicating a central core of caseous necrosis) or exhibit ‘ghost-like’ enhancement within a matted mass of indiscrete nodes [15,16].

Lung parenchymal complications distal to lymph node compression follow a predictable stepwise course [17]. These features can be staged with CT and determine whether lung is salvageable or non-salvageable [17]. Staging can guide medical therapy and determine whether surgical intervention is required, such as enucleation of offending lymph nodes or lobectomy of a non-salvageable lung.

In addition, CT of the chest has the potential to detect features of extrapulmonary TB. The pleura, pericardium, chest wall, spine, and the upper portions of the liver and spleen are visualized in a standard chest CT.

There has been a reluctance for widespread use of CT scanning in children due to the perceived risk of ionizing radiation exposure. Advances in CT imaging techniques, such as the use of extended detector row multi-detector CT scanners, dose modulation, iterative reconstruction, and specific paediatric scanning protocols, have led to a large reduction in ionizing radiation dose [18]. These images can be acquired rapidly without the need for breath holding, reducing the need for procedural sedation and physical constraints [18]. A case report demonstrates the use of low-dose CT scanning to monitor the response to extensively drug resistant TB treatment [19]. The effective dose for each CT in this case was 0.4–0.7 mSv which is equivalent to ±3 months of background radiation exposure [19]. This is much lower than previously documented doses of ± 8.8mSv per scan [20].

Magnetic Resonance Imaging (MRI), although more expensive and less widely available than other imaging modalities, is free from ionizing radiation [2,21,22,23] and is an ideal investigation for follow-up [21,22,23]. MRI long acquisition times in the past required children to have sedation or anesthesia for the procedure. However, the development of rapid MRI sequences, with acquisition times of 10–20s per sequence [23], have made MRI a more attractive investigation, especially in children, which is achievable without sedation [22]. MRI is equivocal to CT in diagnosing primary pulmonary TB in children [23].

## 3. Imaging Findings in Relation to TB Disease

### Primary TB

Following an incubation period, during which the chest radiograph is typically normal, hilar or mediastinal lymphadenopathy occur as part of primary disease (Table 2) [2]. Regional lymphadenopathy is the radiological hallmark of paediatric PTB, with right hilar and right paratracheal regions predominating [24,25]. Nodal enlargement is present in 91–100% of PTB cases in children under 5 years, with the prevalence decreasing with increasing age [25,26].

**Figure 1:**
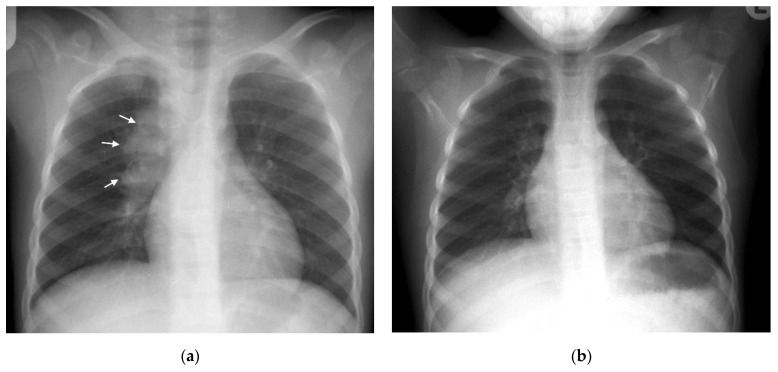
(**a**,**b**): Right Paratracheal and Hilar lymphadenopathy before and after treatment. (**a**) AP chest radiograph of a child at presentation, who later was later confirmed to have pulmonary TB, demonstrates a right-sided lobulated cardio-mediastinal margin with filling of the right hilar point (white arrows) and consistent with right paratracheal and hilar lymphadenopathy. The trachea is displaced to the left, slightly bowed and shows decreased calibre just superior to the carina. There is an oval density seen separately from the scapula in the right lung apex, which in conjunction with the lymphadenopathy, constitutes the Ghon Complex. (**b**) Post-treatment AP chest radiograph demonstrates complete resolution of the parenchymal focus and lymphadenopathy with a normal right cardio-mediastinal border and return of the trachea to its normal shape and position.

**Figure 2:**
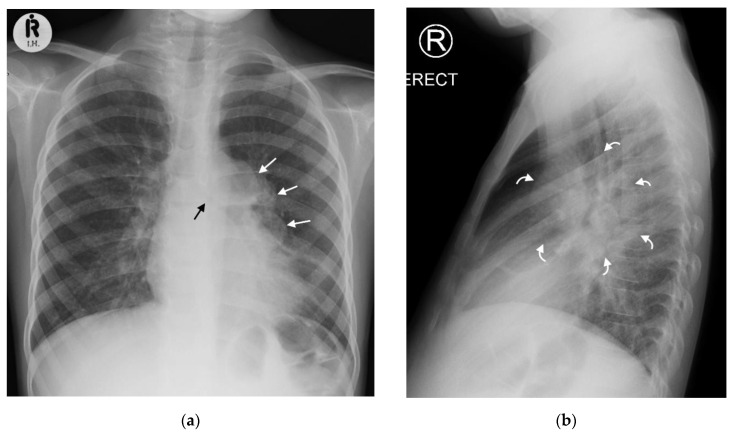
(**a**,**b**): Left hilar lymphadenopathy on the PA and lateral chest radiographs. (**a**) PA erect chest radiograph in this child with later confirmed pulmonary TB demonstrates a multilobulated lymph node mass projecting beyond the cardiac margin on the left (white arrows) consistent with left hilar lymphadenopathy. There is also loss of the left cardiac margin consistent with lingula air-space disease/atelectasis as a consequence of left main bronchus compression (black arrow). (**b**) Lateral chest radiograph confirms the presence of hilar lymphadenopathy by demonstrating an oval mass consistent with the ‘doughnut sign’ (curved white arrows), representing lymphadenopathy inferiorly and likely the normal vessels (aortic arch and left main pulmonary artery) superiorly.

Right hilar adenopathy (a lobulated hilar opacity on chest radiograph) obscuring the hilar point, Figure 1, is more commonly observed than left, with left hilar nodes only evident when extending beyond the left cardiac border [27]. Paratracheal adenopathy may be difficult to distinguish from other mediastinal tissues, such as the thymus, but will extend beyond the normal mediastinal contours, and may result in airway compression and deviation [27]. Left paratracheal lymphadenopathy is rarely observed in isolation, most commonly co-existing alongside other regions of mediastinal lymphadenopathy [27]. Sub- or retro-carinal lymphadenopathy is typically seen on the lateral projection as lobulated densities inferior and posterior to the bronchus intermedius, which, in combination with the more superior aortic arch and pulmonary arteries result in the well described “doughnut sign” (Figure 2).

Ultrasound examination—via the suprasternal notch window—can be used to identify anterior mediastinal lymphadenopathy. These lymph nodes appear as round or oval-shaped, well-defined structures visible in the anterior and superior mediastinum usually surrounded by thymic tissue or mediastinal vessels [28,29]. Lymph nodes are hypoechoic compared with thymic tissue and mediastinal fat and hyperechoic compared with surrounding blood vessels [29]. Ultrasound may also be useful to monitor treatment response [30].

In one study, 67% of tuberculin-skin-test-positive children with normal chest X-rays had enlarged lymph nodes detectable via mediastinal ultrasound [31]. Ultrasound more commonly detects mediastinal lymphadenopathy than plain chest radiographs with superior inter-reader agreement [10]. Despite this, the specificity of enlarged mediastinal lymph nodes is uncertain. Lymph nodes were identified in all groups of children (confirmed TB, suspected TB, and unlikely TB) with significantly larger nodes (>1.1 cm) seen in confirmed and suspected cases over those seen in unlikely TB cases [10]. This suggests the need for an agreed lymph node size cut off to distinguish PTB from other infections.

**Figure 3:**
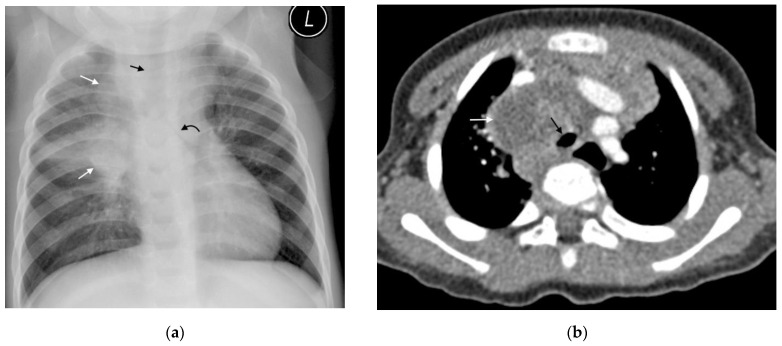
(**a**,**b**): Lymphadenopathy, air-space disease and airway compression on chest radiograph and CT. (**a**) Frontal AP chest radiograph in a 14-month-old boy with confirmed pulmonary TB demonstrating right hilar and paratracheal lymphadenopathy as lobulated masses projecting from the right of the cardio-mediastinal shadow (white arrows). There is also air-space disease in the right upper lobe, tracheal compression (black arrow) and left main bronchus compression (curved black arrow), resulting from presumed subcarinal and left hilar lymphadenopathy. (**b**) Axial post-contrast CT scan confirming the right paratracheal lymphadenopathy which has a low-density centre and fine rim enhancement (white arrow) and AP compression of the trachea (black arrow), which was not appreciated on the AP radiograph.

CT can identify lymphadenopathy in a significant proportion of children with PTB and normal chest radiographs [13]. On post-contrast CT, TB lymphadenopathy typically appears as having low attenuation centrally with peripheral rim enhancement [2] (Figure 3). The central region of low attenuation represents caseous necrotic tissue seen in tuberculous lymphadenopathy, enabling this to be distinguished from non-TB adenopathy. Alternatively, TB nodes may form a matted conglomerate with ‘ghost-like’ rim enhancement [16]. Moderately enlarged lymph nodes may occur in bacterial pneumonia, but rarely having areas of necrosis or calcification [15].

**Figure 4:**
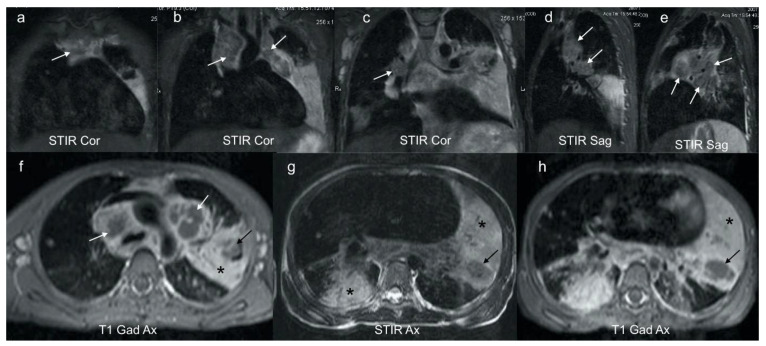
(**a**–**h**)**:** Coronal, sagittal and axial MRI in a 6-year-old girl with confirmed pulmonary TB. Coronal (**a**–**c**) and sagittal STIR (**d**,**e**) images demonstrate characteristic low signal TB lymphadenopathy (white arrows) in the right and left paratracheal, and hilar regions. These can be compared with the higher signal axillary lymph nodes in image (**c**) which represent the appearance of non-TB nodes. Axial post gadolinium T1 at the level of the aortic arch (**f**) demonstrates that the STIR low signal lymphadenopathy in (**a**–**e**) demonstrates rim enhancement (white arrows), typical of centrally necrotic TB nodes. In addition, there is homogenous enhancement of a dense left consolidation (star) with an area of non-enhancing low signal (black arrow), in keeping with parenchymal breakdown within the consolidation. The axial STIR (**g**) and corresponding gadolinium enhanced T1 (**h**) at the lower zone of the lungs demonstrates an intermediate-to-high STIR signal enhancing consolidation posteriorly on the right (star) in keeping with viable lung; an intermediate STIR signal, poorly and heterogeneously enhancing consolidation on the left (star) in keeping with at risk lung; and a T2 low STIR signal non-enhancing focal area on the left (black arrow) typical of TB necrosis (this is the opposite to the STIR signal of an abscess, which would be bright).

MRI is comparable to CT in the detection of lymph nodes >3 mm [22]. However, due to the lower spatial resolution, MRI is unable to detect small lymph nodes <3 mm. Normal lung parenchyma on MRI has a low signal and MRI is poorer at detecting subtle abnormalities such as ground glass opacification and mosaic attenuation [22]. MRI can, however, further differentiate TB lymphadenopathy from reactive lymph nodes based on signal intensity and heterogeneity. The presence of enhancement post contrast suggests active disease [2,3,21]. Short Tau inversion recovery (STIR)/T2-weighted MRI sequences may demonstrate characteristic low signal in TB lymphadenopathy and parenchymal necrosis [2] (Figure 4).

## 4. Primary Progressive TB

### 4.1. Progressive Adenopathy/Lymphotracheobronchial TB

Disruption of airways by tuberculous lymph nodes and the subsequent parenchymal complications define lymphotracheobronchial TB. Younger children are more likely to develop lymphotracheobronchial TB as they have a higher prevalence of lymphadenopathy [25,26], smaller bronchial lumen diameter, and weaker cartilaginous support structures compared with adults. Common complications of nodal compression include air-trapping, atelectasis, consolidation, expansile pneumonia, necrosis, or breakdown [17]. The bronchus intermedius is the most commonly involved, likely because it is longer and narrower than the second order bronchi and is situated between right hilar and sub-carinal nodes [14]. Airway compression is the most reliable chest radiograph feature of lymphadenopathy [5]. The trachea can be displaced, or attenuated by paratracheal lymphadenopathy [5]. On a chest radiograph, compression of airways can result in ipsilateral hyperinflation and atelectasis, or “collapse-consolidation” [1,4].

High-kilovolt (KV) frontal radiographs have been used to better demonstrate the tracheobronchial tree, and the compressive effects of lymphadenopathy in children [4]. However, the addition of high-kV radiographs to standard radiographs has not been shown to significantly increase the sensitivity (38% vs. 38.8%, respectively) or specificity (86% vs. 74.4%, respectively) in detecting PTB amongst patients with microbiologically confirmed TB [4]. Furthermore, the cost of imaging may increase as much as 45% if high-kV imaging is performed [4]. It is thus recommended that high-kV imaging is used to better demonstrate airway attenuation only in select cases of persistent collapse, when CT scans are not readily available [4,5].

**Figure 5:**
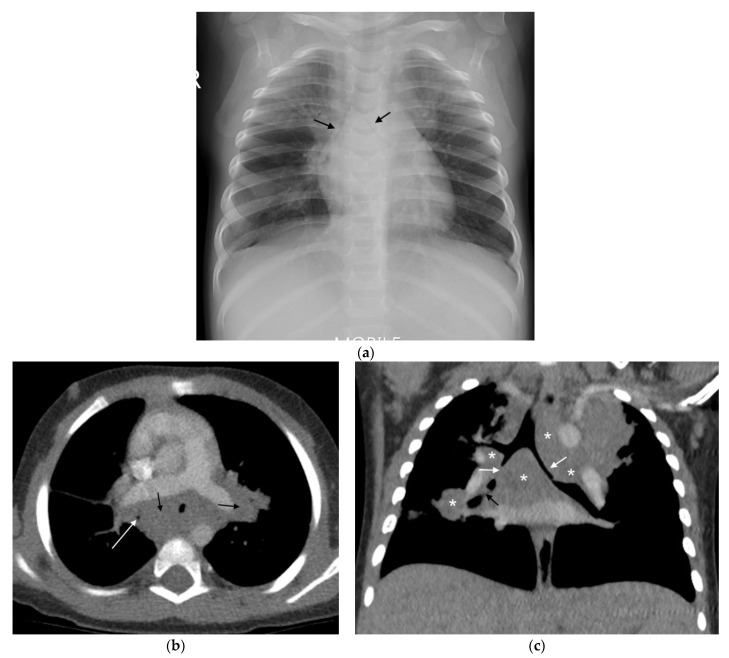
(**a**–**c**)**:** Lymphobronchial TB. Chest radiograph and CT in a 13-month-old boy with confirmed pulmonary TB: (**a**) The frontal AP chest radiograph is suggestive of bilateral hilar and paratracheal lymphadenopathy by the presence of bronchus intermedius and left main bronchus compressions resulting in bilateral (black arrows), mid and lower zone air-trapping. (**b**) Axial, post-contrast, soft-tissue-windowed CT scan at the level of the pulmonary trunk bifurcation demonstrates extensive subcarinal and hilar lymphadenopathy (black arrows) with marked bronchus intermedius attenuation (white arrow). (**c**) Coronal reconstruction of the post-contrast soft-tissue-windowed CT scan demonstrates paratracheal, sub-carinal and hilar lymphadenopathy (stars). There is attenuation of the bronchus intermedius and the left main bronchus (white arrows). There is also a suggestion of erosion of a right hilar lymph node into the lumen of the bronchus intermedius (black arrow).

CT is an excellent tool for assessing the airways and identifying multifocal, segmental areas of air-trapping, atelectasis, and consolidation [14,32]. Additionally, CT can help to determine the cause of tracheobronchial attenuation [1]. Extrinsic compression of a bronchus by an adjacent lymph node causes smooth luminal narrowing. Figure 5. This can create a ball valve phenomenon leading to distal air-trapping. Complete occlusion of the lumen can then result in atelectasis or consolidation and progress to necrosis and cavitation if left untreated [17]. Irregular bronchial lumen narrowing may indicate erosion of an adjacent node into the lumen [1,14].

The anatomic detail of CT allows for accurate assessment of lymph node compression. Multiplanar reconstruction—especially coronal thick slab minimum intensity projection—allows assessment of the large airway in its entirety. Three-dimensional volumetric rendering adds further accuracy with the ability to measure stenosis length and predict whether the offending node is endobronchial, submucosal, or peri-bronchial [32]. These techniques provide a virtual road map for the monitoring of treatment response, identification of complications, bronchoscopy, and precise surgical planning if enucleation of a node is required.

### 4.2. Airspace Disease

**Figure 6:**
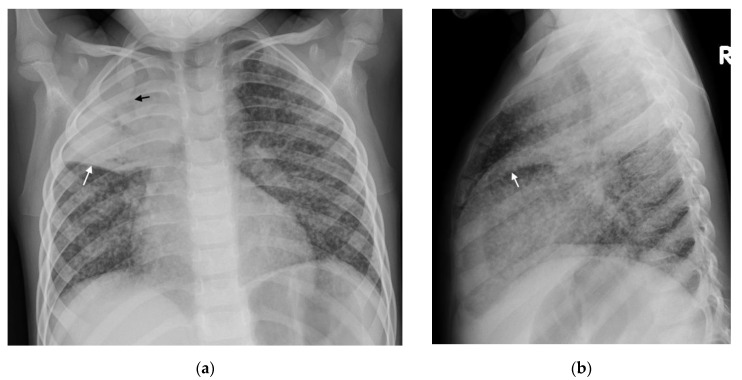
Consolidation and miliary TB. Frontal AP and lateral chest radiographs in a 3-year-old boy with confirmed disseminated TB. This child did not receive BCG vaccination. (**a**) Frontal AP and (**b**) lateral chest radiographs demonstrating right upper lobe consolidation with air bronchograms (black arrow) limited by the horizontal fissure (white arrows), as well as parenchymal miliary TB nodules.

Consolidation can occur via several distinct mechanisms. Firstly, it may represent primary parenchymal PTB disease, which then spreads to regional lymph nodes. Conversely, in primary progressive TB disease, consolidation can develop as a complication of airway compression or from bronchogenic spread of disease [1,14]. Consolidation is characterized on chest radiographs by air bronchograms and silhouetting of the cardiac, mediastinal, or diaphragmatic margins, depending on the location [5] (Figure 6).

The ability to identify peripheral consolidation with ultrasound is comparable to chest radiographs [10]. This is consistent with previous pneumonia studies [33]. Small consolidation (<0.5 cm), usually not identified on chest radiographs, is more frequently seen when using ultrasound [10].

Children over 5 years more commonly exhibit “tree-in-bud” consolidation on CT scan. This represents multiple areas of centrilobular nodules [25]. As the disease progresses, consolidation may develop areas of caseous necrosis centrally—this is represented by areas of low attenuation which do not enhance post-administration of contrast. Rarely, airspace disease progresses to cavity formation, as is commonly seen in adult TB [1,2,25].

MRI is superior to CT in the characterization of tuberculous consolidation in that the MRI signal varies with the stage of necrosis and the presence of mycobacterium within the necrosis [2,23]. Caseous necrosis in TB consolidation demonstrates a characteristic low signal on T2-weighted sequences and is an indicator of active TB [2] [Figure 4].

### 4.3. Miliary TB

Miliary TB is more common in young or immunocompromised patients [2] occurring secondary to hematogenous spread of the disease. It is characterized on the chest radiograph by the diffusion of small (<2 mm) non-calcified nodules, representing granulomas, appearing throughout the lung parenchyma, frequently in combination with thickened interlobular septal lines [1] (Figure 6). However, in 25–40% of cases, chest radiographs are normal [1]. Miliary nodules occur on CT well before they become visible on chest radiographs [1].

## 5. Post Primary TB

### Cavitation

**Figure 7:**
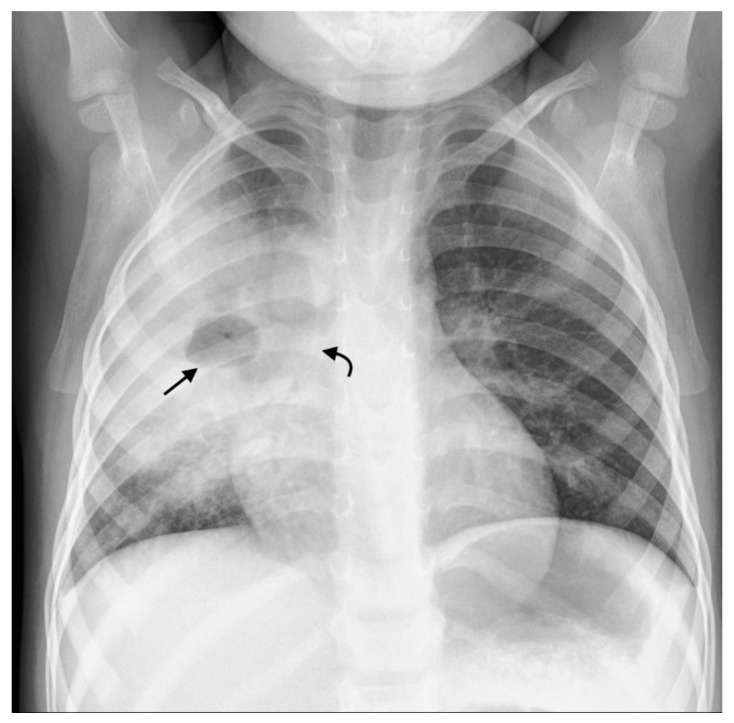
Frontal PA chest radiograph in a 3-year-old boy with confirmed pulmonary TB demonstrating right middle zone consolidation and cavitation (straight black arrow) containing an air-fluid level, as well as narrowing of the bronchus intermedius (curved black arrow).

Post-primary cavitation is seen more commonly in adolescents and is the hallmark of post-primary TB on chest radiographs (Figure 7). Cavitation may also result from nodular attenuation of bronchi or progressive primary disease, with the development of multiple bilateral cavities in younger children who are usually very ill [1,2]. The chest radiograph in the latter typically demonstrates air space consolidation and cavitation (resulting from caseous necrosis and liquefaction) in the upper lobes, and apical segments of the lower lobes. Air-fluid levels, usually resulting from secondary infection, may occur. These may be associated with multiple micronodules within a lobe or segment, representing post-primary bronchogenic spread [1].

CT is not only superior in differentiating consolidation from cavitation, when compared with chest radiography, but it provides the ability to describe the cavity wall morphology [1]. MRI is as sensitive as CT in the detection of cavities [22].

## 6. Complications

### 6.1. Bronchiectasis

Parenchymal destruction and chronic fibrosis can lead to traction bronchiectasis [32]. Changes on chest radiograph may be subtle. Grossly dilated bronchial lumens and thickened bronchial walls lead to increased bronchovascular markings and bronchi-imaged end ons appear as ring shadows.

CT is much more sensitive for demonstrating bronchiectasis. The ‘tram track sign’ represents thickened non-tapering bronchi. An increased broncho-arterial ratio, >0.8 in children [34], is indicative of bronchiectasis. When cut in a cross-section, the dilated bronchi and smaller adjacent artery form the classic ‘signet ring’ sign [5].

### 6.2. Pleural Disease

**Figure 8:**
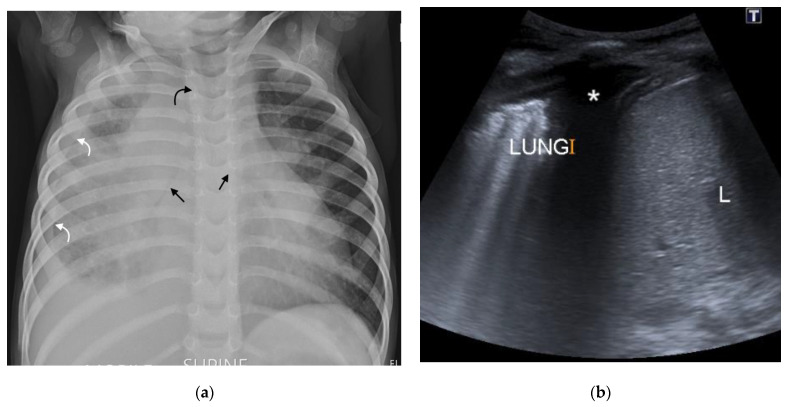
(**a**,**b**): (**a**) Supine AP chest radiograph in a 17-month-old male with PTB demonstrating right sided pleural effusion with veiling of the right hemi-thorax and a lamellar component tracking up along the lateral chest wall (curved white arrows). There is also attenuation of the bronchi bilaterally (straight black arrows), bowing of the trachea from lymphadenopathy (curved black arrow) and an enlarged, globular-shaped heart, consistent with a pericardial effusion (confirmed on ultrasound, not shown here). (**b**) Chest ultrasound confirming the pleural effusion (star) seen as hypoechoic fluid between the parietal pleura, diaphragm, and right lung.

Pleural disease occurs via either of two mechanisms: direct spread from a caseating sub-pleural focus (consolidation or lymph node) or via hematogenous spread [1]. Pleural disease is more common in older children [2]. A pleural effusion may occur secondary to an obstruction of lymphatic drainage or as a result of a hypersensitivity reaction. This explains why most pleural fluid cultures are negative [1]. They are typically unilateral, lamellar (a linear density extending along the lateral chest wall and sparing the costophrenic angle), and associated with consolidation or lymphadenopathy [3,6] (Figure 8). A large volume of pleural fluid needs to collect before becoming visible on plain films [2].

A simple pleural effusion on ultrasound is characterized by an anechoic fluid collection separating the visceral and parietal pleura. Ultrasound has been shown to be more sensitive than a chest radiograph for the presence of a tuberculous pleural effusion [10]. An added benefit of ultrasound is the ability to further characterize the effusion by detecting the presence of loculations or empyema as well as guide drainage of these effusions if needed.

CT and MRI have the ability to quantify the size of a pleural effusion and differentiate pleural thickening from an effusion. MRI is more sensitive than non-contrast CT for pleural abnormalities [2,23], and MRI can better delineate internal debris and septations in pleural effusions [2,22].

The ‘split pleura’ sign on CT—linear smooth enhancing visceral and parietal pleura encasing a loculated collection—represents empyema [1,5]. Empyema is a non-specific finding most commonly occurring secondary to bacterial pneumonia; however, *M. tuberculosis* remains an important cause in high-burden regions [35]. Empyema can be complicated by fistula formation into the subcutaneous space or the bronchopulmonary tree [1].

### 6.3. Pericardial Disease

TB pericarditis occurs due to the erosion of lymph nodes into the pericardium but may also occur due to hematogenous dissemination. Chest radiograph typically demonstrates an enlarged and globular cardiac silhouette [1,5]. In pericardial disease, a CT scan will demonstrate pericardial thickening, with or without an effusion, as well as regional lymphadenopathy. The pericardial sac may be fibrosed or calcified, which can result in constrictive pericarditis.

## 7. New Imaging Techniques/Technology

Dynamic 4-D CT scans allow for 3D volumetric rendering of the airways for accurate measurement of bronchial stenosis length and could provide a safer non-invasive alternative to bronchoscopy to assess tracheobronchomalacia (Table 3) [18]. The anatomic detail provided by CT can help to determine the underlying cause for the stenosis and ultimately guide intervention [18]. However, CT scanners with volume scanning capabilities are not widely available and the (unfounded) perception that they impart a higher radiation dose than bronchography limits its use [18].

Newer MRI techniques can provide information regarding lung perfusion and ventilation [23]. High cost of hyperpolarized gas and dedicated hardware for lung ventilation MRI poses a challenge [23]. Fourier decomposition is a new experimental technique that can provide ventilation and perfusion images in a single acquisition, and recent tests have provided information on hyperpolarized gas MRI and contrast-enhanced MRI [23].

Priftakis et al. demonstrated that positron emission tomography (PET)/CT with 18F-fluorodeoxyglucose (FDG) is highly sensitive in active TB and has shown potential in the early detection of TB as well as in the assessment of the response to treatment [27]. However, PET/CT with FDG has been shown to have a low specificity in the setting of solitary pulmonary nodules with a poor ability to differentiate TB from malignancy. Availability is also limited. [27]

In the last two decades, computer-aided detection (CAD) software has been developed to independently locate and define radiological abnormalities on chest radiographs and improve the sensitivity and specificity, by predicting the likelihood of TB disease based on scoring systems [36]. This software has been intended primarily for high-burdened, low-middle income countries with limited access to radiologists. It has been shown to be cost-effective and user friendly [37,38]. In March 2020, the WHO endorsed the use of CAD software as an alternative to human interpretation of chest radiographs in TB screening and triage amongst susceptible adults, over the age of 15 years, after three commercially available software met WHO criteria for the minimal acceptable sensitivity (90%) and specificity (70%) of a TB triage test, when compared with GeneXpert or culture [36].

More recently, five commercially available artificial intelligence programs were analysed using chest radiographs from 23,954 adults from Bangladesh presenting for TB screening. All five algorithms were shown to significantly outperform experienced radiologists in detecting abnormalities associated with PTB, with sensitivities above 90%. Furthermore, they resulted in a 50% reduction in the need for GeneXpert testing [37]. Sensitivities varied between patient populations and contexts, with lower values in participants who were older, and those who had previous TB [37].

Despite these promising data in adults, the literature regarding the performance of CAD in diagnosing primary TB and its complications in children is sparse, and further research is required [37].

## 8. Conclusions

Chest imaging plays a crucial role in the diagnosis and management of pediatric PTB. Chest radiography remains the primary investigation for the assessment of PTB, especially in high burden areas. However, for lymphadenopathy, the cardinal sign of primary disease, chest radiography has low sensitivity and specificity. The use of ultrasound, CT, or MRI can augment diagnostic ability, which can improve case detection. Newer imaging techniques such as dynamic 4D CT, (PET)/CT, and CAD software may improve radiological accuracy and help guide intervention.

## Figures and Tables

**Table 1:** Advantages and disadvantages of commonly available imaging modalities.

Imaging Modality	Advantages	Disadvantages
Chest radiograph	Widespread availability Low dose ionizing radiation Cost effective	Poor intra- and inter-observer agreement Poor sensitivity/specificity
Ultrasound	Performed at bedside	Requires user experience
	Free of ionizing radiation Detects mediastinal nodes or pleural effusion before CXR Ability to assess for extrapulmonary TB	Sensitivity/specificity data for signs still scanty Unable to assess pulmonary hila for lymphadenopathy
Computed tomography (CT)	Earlier more sensitive detection of TB disease and complications compared with CXR	Expensive Requires specific expertise
	Ability to monitor disease complications and treatment response	Ionizing radiation although low dose protocols now in use
	Higher sensitivity for detecting nodes Allows for surgical planning Characterization of lymph node morphology and enhancement May differentiate TB from non-TB lymphadenopathy	Limited availability May require contrast
Magnetic resonance Imaging (MRI)	Sensitivity/specificity comparable to CT (except small nodules/GGO) Differentiate TB lymphadenopathy from reactive lymph nodes based on signal intensity and heterogeneity	Expensive Requires specific expertise Limited availability May require sedation/anesthesia Longer scanning times (relative to other modalities)

**Table 2:** Imaging findings for each form of PTB.

Form of PTB	Imaging Findings	Comments
**Primary TB**	***Lymphadenopathy*** CXR: Lobulated hilar/paratracheal opacity. Potential for airway attenuation or deviation. Doughnut sign on lateral radiograph. US: Well defined round/oval hypoechoic (to thymic tissue and fat) nodes within the anterior and superior mediastinum. CT: Typically, low attenuation centrally with peripheral rim enhancement of node post contrast administration. Alternatively, matted conglomerate with ‘ghost-like’ rim enhancement. MRI: Low T2/STIR signal intensity nodes. Post gadolinium T1 images may demonstrate rim enhancement.	Right sided lymphadenopathy more common than left. CXR typically normal during incubation period. US unable to assess hilar region. CT detects nodes in a significant proportion of patients with normal CXR. Central low attenuation with peripheral enhancement helps distinguish from non-TB adenopathy. MRI comparable to CT in node detection over 3 mm.
**Primary** **progressive TB**	***Progressive adenopathy*** CXR: Airway compression or displacement most reliable finding. Attenuation can result in distal ipsilateral hyperinflation, atelectasis or consolidation. US: Unable to assess airway compression but may detect distal complications. CT: Smooth luminal narrowing indicates extrinsic compression. Irregular narrowing may indicate erosion into lumen. Excellent for identifying complications, planning treatment and monitoring treatment response. MRI: Detection of compressive nodes and distal complications comparable to CT. Poorer resolution (in comparison with CT) makes airway lumen assessment and exact nodal location identification difficult. ***Airspace disease*** CXR: Opacification of lung parenchyma silhouetting adjacent structures. May display air bronchograms. US: Comparable detection rates to CXR with peripheral consolidation. Able to identify <0.5cm consolidation (usually undetectable on CXR). CT: Classic ‘tree-in-bud’ pattern. Central low attenuation non-enhancing regions represent caseous necrosis. MRI: Able to characterize TB consolidation. Consolidation in viable lung tissue demonstrates intermediate-to-high STIR signal. Low signal on STIR sequence indicates necrotic lung tissue. ***Miliary TB***	Younger children more likely to develop nodal airway compression due to inherently narrower airways and weaker cartilaginous support structures. Airway attenuation is the most reliable CXR sign. Distal complications of airway compression include atelectasis, air-trapping, consolidation, necrosis and breakdown. Airway attenuation and characterization of complications better characterized by CT and MRI. Miliary TB best identified by presence of diffuse small nodules and thickened septal lines. CT is the superior imaging technique.
	CXR: Often normal. Diffuse small non-calcified nodules. Thickened interlobular septal lines. US: No sensitive findings in children yet described. CT: Miliary nodules visualized well before visible on CXR. Small (<3 mm) randomly distributed nodules with thickened interlobular septa. MRI: Unable to detect <3 mm nodules. Useful in detecting lesions in solid organs (liver/spleen)	
**Post primary TB**	***Cavitation*** CXR: Often difficult to distinguish small cavity from consolidation. Airspace opacification surrounding an area of cavitation represents central caseous necrosis and liquefaction. Air-fluid level may represent secondary infection. CT: Central low-attenuating cavity. Cavity wall variable in size. Cavity surrounded by consolidation. MRI: Low signal cavity with surrounding consolidation.	Cavity formation is the hallmark of post-primary TB. Small cavities easily missed on CXR. CT and MRI superior to CXR in the detection of cavities. Usually predominate in upper lobes or apical segments of lower lobes. More common in adolescents. CT is useful in assessing cavity wall thickness.

**Table 3:** Advantages and disadvantages of future imaging modalities.

Imaging Modality	Advantages	Disadvantages
***Dynamic 4-D CT scans***	Accurately demonstrates tracheobronchomalacia. Demonstrates structures adjacent to the tracheobronchial tree. Non-invasive. Fast. Allows for 3D reconstruction.	Limited availability. Perceived to impart a higher radiation dose than bronchography.
***Newer MRI techniques***	Provide ventilation and perfusion images in a single acquisition. Shorter acquisition times. No radiation exposure.	High cost. Limited availability.
***Positron emission*** ***tomography (PET)/CT***	Highly sensitive in active TB. Reliably differentiates between active and latent disease. Assists with assessing response to treatment.	Limited availability. Low specificity with solitary pulmonary nodules.
***Computer aided*** ***detection software (CAD)***	Acceptable sensitivity (90%) and specificity (70%) of a TB triage test. Cost-effective. User friendly. No human expertise needed to interpret	Sparse literature regarding performance in paediatrics. Lower sensitivities in older patients and those with previous TB.

## Data Availability

Not applicable.
